# The Association Between Polymorphisms in Cell-Cycle Genes and Mitochondrial DNA Copy Number in Coke Oven Workers

**DOI:** 10.3389/fpubh.2022.904856

**Published:** 2022-07-05

**Authors:** Yuping Wang, Jiebing Tan, Wei Wang, Xiaoran Duan, Brooke Lappe, Liuhua Shi, Yongli Yang, Xuezhong Shi

**Affiliations:** ^1^Department of Epidemiology and Biostatistics, College of Public Health, Zhengzhou University, Zhengzhou, China; ^2^Department of Occupational Health and Occupational Diseases, College of Public Health, Zhengzhou University, Zhengzhou, China; ^3^Internet Medical and System Applications of National Engineering Laboratory, The First Affiliated Hospital of Zhengzhou University, Zhengzhou, China; ^4^Gangarosa Department of Environmental Health, Rollins School of Public Health, Emory University, Atlanta, GA, United States

**Keywords:** mitochondrial DNA copy number, coke oven emissions, cell-cycle genes, polymorphisms, generalized linear models (GLM)

## Abstract

The mitochondrial DNA (mtDNA) copy number is a vital component in maintaining normal mitochondrial function. It is affected by environmental and occupational exposures, as well as polymorphisms in nuclear genes. Nonetheless, the specific roles of polymorphisms in cell-cycle genes and mtDNA copy number are still unknown. This study enrolled a sample of 544 coke oven workers and 238 non-exposed controls so as to assess the effect of exposure of coke oven emissions (COEs) and polymorphisms in cell-cycle genes on the mtDNA copy number. We found that the mtDNA copy number in the exposed group (0.60 ± 0.29) was significantly lower than that in the control group (1.03 ± 0.31) (*t* =18.931, *P* < 0.001). The analysis of covariance showed that both the rs1801270 (CA+CC) and the rs1059234 (CT+CC) in *p21* gene were associated with lower mtDNA copy number in the exposed group (*P* = 0.001). Generalized linear models indicated COEs-exposure (β = −0.432, *P* < 0.001) and rs1059234 (CT+CC) in *p21* gene (β = −0.060, *P* = 0.024) were the factors in mtDNA copy number reduction. In conclusion, this study suggests that the decrease of the mtDNA copy number is associated with COEs-exposure and the rs1059234 (CT+CC) in the *p21* gene.

## Introduction

Mitochondria are subcellular organelles that sustain life through adenosine triphosphate (ATP) generation. Mitochondria also participate in various vital physiological processes such as calcium homeostasis, generation of free radicals, cell apoptosis, among others (1). Additionally, mitochondria uniquely possess their own genome, known as mitochondrial DNA (mtDNA), that present variable copy numbers in cells based on surroundings the mitochondria inhabit (2). In brief, mtDNA copy number is affected by exogenous and genetic factors.

Coke oven emissions (COEs) are major environmental and occupational hazard factors and contain a variety of toxic substances, including polycyclic aromatic hydrocarbons (PAHs) (3). It is well-established that metabolites of PAHs have carcinogenic and genotoxic potential; long-term exposure can cause the formation of DNA adducts, eventually leading to DNA damage (4). Our previous studies have shown that exposure to PAHs can lead to the decrease in antioxidant capacity and telomere damage (5, 6). If subjected to exogenous stimulation, mtDNA is particularly susceptible to damage due to greater vulnerability to oxidative stress and limited DNA repair capacity (7). To further investigate this process, our lab explored the effects of exposure to PAHs from coke stoves on mtDNA (8).

MtDNA replication is a continuous process which requires coordinated action of several mitochondrial functioning proteins. In addition to nuclear genes encoding functional mitochondrial proteins, some cell-cycle genes influence mtDNA copy number by regulating certain mitochondrial-related genes. The gene *p53* contributes to the regulation of mitochondrial transcription factor A (TFAM) and peroxisome proliferator-activated receptor gamma coactivator 1-alpha (PGC-1alpha), which could modulate replication and transcription of mtDNA (9, 10). Moreover, *p53* also plays an important direct role in the control of mitochondrial genome stability or mtDNA copy number checkpoint (11). *MDM2*, a negative regulator of *p53*, may also control mtDNA copy number through inhibiting *p53* expression (12). The gene *p21* maintains mtDNA integrity by regulating antioxidant enzymes and thus controlling ROS levels (13). And *p21* could modulate mitochondrial biogenesis by controlling the expression of other genes through its transcriptional regulatory function (14). Additionally, several genome-wide association studies have shown that the mtDNA copy number was related to polymorphisms in some nuclear genes (15–17). Our previous study found that the mtDNA copy number was associated with miR-210 rs11246190 in coke oven workers (8). However, the association between mtDNA copy number and polymorphisms in cell-cycle genes has not yet been examined. Therefore, this study aims to further explore the association between polymorphisms in cell-cycle genes and the mtDNA copy number in coke oven workers.

## Materials and Methods

### Study Subjects

A total of 782 participants were enrolled in the study, comprising 544 in exposed group and 238 in control group. The coke factory workers in Henan, China, exposed to COEs for more than 1 year were included in exposed group. The healthy participants with no history of exposure to occupational PAHs or other toxicants in the same city during the same period were included in control group. And the participants with liver or kidney diseases or other chronic diseases were excluded from the study. Demographic characteristics, occupational exposure level, and blood samples from all subjects were collected by trained interviewers. The study was approved by the Ethics Committee in Zhengzhou University (IRB 00006861, FWA00014064), and written informed consent was obtained from all subjects.

### COEs-Exposure Levels Assessment

The air samples were collected at four coking plants. Five representative sampling sites were chosen from each coking plant, including the furnace top, furnace side, hearth, duty room and office. We obtained the time-weighted average (TWA) concentration (mg/m^3^) and working time (year) of each participant according to the onsite detecting results and detailed occupational exposure history. Cumulative exposure concentration of COEs (mg/m^3^·year) was used to assess COEs-exposure levels, which is equal to the product of the TWA concentration (mg/m^3^) and working time (year) in the exposed group. The detailed protocol for measurement of COEs-exposure concentration was described in our previous research (18). The cumulative exposure concentration of COEs in the control group was estimated based on their living environmental concentration and age.

### mtDNA Copy Number Detection

The mtDNA copy number of genomic DNA from whole peripheral blood samples was measured using real-time quantitative polymerase chain reaction (PCR) assay by determining the ratio of the mitochondrial gene ND-1 (mtND1) to the nuclear reference gene human β-globin (HBG) (19). Table 1 shows primer sequences of the two genes. The PCR was conducted using the 2 × SYBR Green Master Mix, and was carried out in the following steps: an initial heating step of 95°C for 5 min was followed by 40 cycles of 10 s at 95°C for denaturation and 30 s at 60°C for annealing/extension. Each sample was run using two parallels. Relative mtDNA copy number was calculated using threshold cycle (Ct) values and the formula of 2^−ΔΔCt^ (ΔCt = Ct_mtDNA_-Ct_Reference_, ΔΔCt = ΔCt-ΔCt_averagenormalcontrols_).

**Table 1:** Primers sequences for Real-time PCR.

**Gene**	**Primers sequences**
*mtND1*	Forward: 5′-CCTAATGCTTACCGAACGA-3′
	Reverse: 5′-GGGTGATGGTAGATGTGGC-3′
*HBG*	Forward: 5′-GCTTCTGACACAACTGTGTTCACTAGC-3′
	Reverse: 5′-CACCAACTTCATCCACGTTCACC-3′

*MtND1, Mitochondrial gene ND-1*.

*HBG, Human β-globin*.

### Genotyping Analysis

Genomic DNA was isolated from whole peripheral blood samples in order to conduct genotyping. This study focused on important functional gene polymorphisms and supplemented susceptible gene polymorphisms, and screened six polymorphic loci in three genes (*p53* rs17878362, *p53* rs1042522, *p53*rs1625895, *p21* rs1801270, *p21*rs1059234, and *MDM2*rs3730485). Polymorphisms in *p53* rs17878362 and *MDM2* rs3730485 were detected by PCR directly, and other loci were genotyped by the PCR-restriction fragment length polymorphism (PCR-RFLP) method. The details of genotyping, primer sequences, restriction endonucleases, and PCR have been reported in previous research (20–22).

### Statistical Analysis

Statistical analysis was conducted by SPSS 27.0 software (SPSS Inc., Chicago, IL, USA). The student *t*-test was performed to compare the differences between the exposure group and the control group for mtDNA copy number showed a normal distribution. Partial correlations or the analysis of variance (ANOVA) was conducted to explore the association between cumulative exposure dose or individual characteristics and mtDNA copy number. The effects of gene polymorphisms on the mtDNA copy number were examined using the analysis of covariance (ANCOVA), and the Least Significant Difference test was used to conduct the pairwise comparisons. Generalized linear models (GLMs) were applied to analyze the environment, gene, and interaction on mtDNA copy number (23). The genotype distribution in the control group was tested with Hardy-Weinberg equilibrium. Linkage disequilibrium analysis was tested online based on the method described by Shi and He (24). All statistical tests were two-sided, and the level of statistical significance was set at *a* = 0.05.

## Results

### Population-Based Data

Comparative analysis of cumulative exposure dose and individual characteristics including gender, age, smoking status, drinking status, and body mass index (BMI), between the exposed group and the control group can be seen from our previous research (18). The results showed that the differences between the exposed group and the control group were significant (*P* < 0.05) in terms of cumulative exposure dose [1.12 (0.34, 2.14) mg/m^3^·year vs. 0.07 (0.06, 0.09) mg/m^3^·year], male (71.7 vs. 58.4%), age (40.10 ± 6.30 a vs. 38.39 ± 8.43 a), smoking (41.0 vs. 17.2%), and drinking (54.4 vs. 42.0%).

### mtDNA Copy Number and COEs-Exposure

The mtDNA copy number in the exposed group (0.60 ± 0.29) was lower than in the control group (1.03 ± 0.31) (*t* = 18.931, *P* < 0.001). Partial correlations showed that the mtDNA copy number was negatively correlated with cumulative exposure dose (r = −0.253, *P* < 0.001) after controlling for gender, age, smoking status, drinking status and BMI.

### mtDNA Copy Number and Individual Characteristics

As reported in our previous research (25), the results were shown in Table 2. The effect of gender on mtDNA copy number was statistically significant in the control group but not in the exposed group. The effect of age, smoking status, drinking status and BMI on mtDNA copy number was not found in either the exposed group or the control group (*P* > 0.05). And the effect of working time was not significant in exposed group (*P* = 0.118). The differences of mtDNA copy number between the exposed group and the control group were also observed in subgroups, and the analysis showed that the mtDNA copy number between the exposed group and the control group had significant differences between each subgroup.

**Table 2:** MtDNA copy number by individual characteristics.

**Variables**	**Exposure**			**Control**			***P* ^b^**
	***n***	**mean ±SD**	***P* ^a^**	***n***	**mean ±SD**	***P* ^a^**	
Gender							
Male	390	0.59 ± 0.30	0.201	139	1.00 ± 0.28	0.039	<0.001
Female	154	0.62 ± 0.29		99	1.08 ± 0.34		<0.001
Age (years)							
≤ 40	273	0.60 ± 0.28	0.971	142	1.05 ± 0.31	0.273	<0.001
>40	271	0.60 ± 0.31		96	1.01 ± 0.30		<0.001
Smoking status							
No	321	0.60 ± 0.29	0.685	197	1.04 ± 0.32	0.364	<0.001
Yes	223	0.59 ± 0.29		41	0.99 ± 0.27		<0.001
Drinking status							
No	248	0.61 ± 0.30	0.167	100	1.05 ± 0.33	0.349	<0.001
Yes	296	0.58 ± 0.29		138	1.01 ± 0.27		<0.001
Body mass index (kg/m^2^)							
<18.5	12	0.70 ± 0.29	0.351	5	1.05 ± 0.22	0.778	0.028
18.5–24.0	224	0.61 ± 0.30		108	1.01 ± 0.33		<0.001
24.0–28.0	230	0.58 ± 0.28		101	1.06 ± 0.29		<0.001
≥28.0	78	0.58 ± 0.33		24	1.03 ± 0.30		<0.001
Working time (years)							
<17	177	0.60 ± 0.27	0.118				
17–25	277	0.57 ± 0.30					
≥25	90	0.65 ± 0.31					

*MtDNA, Mitochondrial DNA*.

*^a^The analysis of variance was adopted to analyze the effects of individual characteristics on mtDNA copy number in exposure group or in control group*.

*^b^The t test was performed to compare the mtDNA copy number between exposure group and control group in subgroups*.

### mtDNA Copy Number and Polymorphisms in Cell-Cycle Genes

The genotype distribution for the six polymorphisms in cell-cycle genes did not deviate from Hardy-Weinberg equilibrium (*P* > 0.05). Table 3 presents the differences of mtDNA copy number between genotypes in polymorphisms. The number of homozygous GG genotype group in *p53* rs1625895 was 0 in the exposed group and 1 in the control group. Consequently, they were merged with the heterozygous AG genotype group for statistical analysis. Due to the mtDNA copy number in the heterozygous CA genotype group in *p21* rs1801270 being similar to that in the wild homozygous genotype CC group, the CA genotype group was merged with CC group. The mtDNA copy number in the CA+CC group in *p21* rs1801270 was statistically lower than in the mutation homozygous AA genotype group in the exposed group. For the same reason, the heterozygous CT genotype group in *p21* rs1059234 was also merged with the wild homozygous genotype CC group. The mtDNA copy number in the CT+CC group in *p21* rs1059234 was statistically lower than in the mutation homozygous TT genotype group in exposure group.

**Table 3:** MtDNA copy number by individual polymorphisms in cell-cycle genes.

**Polymorphisms**	**Exposure**			**Control**			***P* ^b^**
	***n***	**mean ±SD**	***P* ^a^**	***n***	**mean ±SD**	***P* ^a^**	
***p53*** **rs17878362**							
D/D	502	0.60 ± 0.29	Ref.	216	1.04 ± 0.31	Ref.	<0.001
D/I	42	0.54 ± 0.29	0.186	22	1.00 ± 0.28	0.393	<0.001
***p53*** **rs1042522**							
CC	128	0.60 ± 0.29	Ref.	54	1.01 ± 0.28	Ref.	<0.001
CG	252	0.58 ± 0.28	0.532	115	1.05 ± 0.32	0.257	<0.001
GG	164	0.61 ± 0.31	0.920	69	1.03 ± 0.31	0.656	<0.001
***p53*** **rs1625895**							
AA	483	0.60 ± 0.29	Ref.	213	1.03 ± 0.32	Ref.	<0.001
AG+GG	61	0.59 ± 0.30	0.991	25	1.04 ± 0.24	0.916	<0.001
***p21*** **rs1801270**							
AA	115	0.66 ± 0.28	Ref.	54	1.05 ± 0.28	Ref.	<0.001
CA+CC	429	0.58 ± 0.30	0.006	184	1.03 ± 0.32	0.990	<0.001
***p21*** **rs1059234**							
TT	112	0.66 ± 0.28	Ref.	46	1.03 ± 0.33	Ref.	<0.001
CT+CC	432	0.58 ± 0.30	0.006	192	1.03 ± 0.30	0.719	<0.001
***MDM2*** **rs3730485**							
I/I	263	0.62 ± 0.29	Ref.	118	1.06 ± 0.30	Ref.	<0.001
I/D	233	0.57 ± 0.30	0.124	97	0.99 ± 0.29	0.203	<0.001
D/D	48	0.58 ± 0.26	0.505	23	1.09 ± 0.41	0.540	<0.001

*Ref., The reference group of pairwise comparison, using Least Significant Difference test*.

*MtDNA, Mitochondrial DNA*.

*^a^The covariance analysis was applied to analyze the difference of mtDNA copy number between genotypes, adjusted for gender, age, smoking status, drinking status, and body mass index*.

*^b^The t test was performed to compare the mtDNA copy number between exposure group and control group in genotype subgroups*.

### mtDNA Copy Number and the Interaction of COEs-Exposure and the CA+CC Genotypes in *p21* rs1059234

Linkage disequilibrium analysis was used to examine the connection between rs1801270 and rs1059234 in *p21* gene and showed the D' value was 0.873. This suggests that there is a strong linkage disequilibrium between the two loci. Therefore, we analyzed the effects of the two loci on the mtDNA copy number by focusing on rs1059234 in *p21* gene.

Table 4 summarizes the combined effect of COEs and *p21* rs1059234 on mtDNA copy number. The results of the GLMs showed that the combined effect of COEs-exposure and the CT+CC genotypes in *p21* rs1059234 on the mtDNA copy number was statistically significant, whereas the interaction of the two factors on the mtDNA copy number was not statistically significant [β (95% CI) = −0.096 (−0.209,0.018), x^2^ = 2.723, *P* = 0.099].

**Table 4:** Combined effect of p21 rs1059234 and COEs on mtDNA copy number.

***p21* rs1059234 (CT+CC)**	**COEs-exposure**	**MtDNA copy number (mean ±SD)**	**β (95% CI)**	**x^**2**^**	***P***
–	–	1.03 ± 0.33	Ref.		
+	–	1.03 ± 0.30	0.008 (−0.088, 0.103)	0.027	0.870
–	+	0.66 ± 0.28	−0.355 (−0.457, −0.252)	46.066	<0.001
+	+	0.58 ± 0.30	−0.443 (−0.533, −0.352)	91.640	<0.001

*COEs, Coke oven emissions*.

*MtDNA, Mitochondrial DNA*.

*Ref., The reference group*.

*Adjusted for gender, age, smoking status, drinking status and body mass index*.

### Influencing Factors of mtDNA Copy Number

GLMs were also used to screen influencing factors, using the mtDNA copy number as the dependent variable, COEs and rs1059234 in *p21* gene as predicators, and gender, age, smoking status, drinking status, and BMI as covariates. The variables kept in the model included COEs-exposure (β = −0.432, *P* < 0.001) and rs1059234 (CT+CC) (β = −0.060, *P* = 0.024) (Table 5).

**Table 5:** Influencing factors of mtDNA copy number.

**Parameter**	**β (95% CI)**	**x^**2**^**	***P***
Constant	1.154 (0.951,1.357)	124.270	<0.001
COEs-exposure	−0.432 (−0.478, −0.385)	331.447	<0.001
*p21* rs1059234 (CT+CC)	−0.060 (−0.111, −0.008)	5.068	0.024

*COEs, Coke oven emissions*.

*MtDNA, Mitochondrial DNA*.

*Adjusted for gender, age, smoking status, drinking status and body mass index by generalized linear models*.

## Discussion

MtDNA copy number, as an index of mitochondrial biogenesis, is vital for maintaining normal mitochondrial function and energy production in the body, and it is influenced by both exogenous and genetic factors. In the present research, we demonstrated that COEs exposure, polymorphisms in *p21* and their combined effect significantly affected the alteration of mtDNA copy number.

Emerging epidemiological evidence suggests that exogenous factors and occupational exposures may be related to alterations of mtDNA copy number. The gender (26), age (27), smoking (28), drinking and BMI (29) have been reported to be associated with mtDNA copy number. Our results also showed that the mtDNA copy number was different in male vs. female in the control group. However, we did not observe the significant effect of working time on mtDNA copy number. Possible reasons are as follows, the exposure level varies in five representative sampling sites and workers may have different exposure due to job transfer. Since total COEs-exposure levels is a comprehensive indicator of working time and working location, neither one of these can reflect exposure level alone. Moreover, several researches have shown that benzene and black carbon (19) could cause an increased mtDNA copy number and conversely, lead (30), elemental carbon and arsenic (31) could lead to a decreased mtDNA copy number. PAH, a known carcinogen and mutagen, has also been reported to be related with changes in mtDNA copy number, although the relationships are inconsistent. Pavanello, Dioni (32) found that mtDNA copy number in peripheral blood lymphocytes was higher among coke oven workers with exposure to PAHs. However, more studies reported lower mtDNA copy number in PAH-exposed individuals. Pieters, Koppen (33) observed that blood mtDNA copy number was inversely associated with indoor exposure to PAHs in house dust. Ling, Zhang (34) found an association between decreased sperm mtDNA copy number and PAH exposure in a prospective cohort “Male Reproduction Health in Chongqing College Students.” Our previous study showed that mtDNA copy number in the COEs-exposed group was lower than that in the control group (8). PAHs, the main harmful component of COEs, can cause ROS-induced mtDNA damage. Cells challenged with ROS synthesize more copies of mtDNA and increase the number of mitochondria to compensate for the damage. This causes a vicious cycle to develop over time. The increasing abundance of mitochondria causes the overproduction of ROS and further oxidative damage, which may initiate cell senescence or death, eventually resulting in the decreased mtDNA copy number (19).

In addition, the polymorphisms in cell-cycle genes could affect mtDNA copy number. The genes *p53* and *p21* participate in cell cycle arrest in G1/S transition upon DNA damage and regulate mitochondrial biogenesis. It has been shown that polymorphisms in these cell-cycle genes could affect gene transcription or protein expression, and then modulate gene functions, which consequently may affect mitochondrial biogenesis. Therefore, we screened six polymorphic loci in cell-cycle genes (*p53* rs17878362, *p53* rs1042522, *p53* rs1625895; *p21* rs1801270, *p21*rs1059234, and *MDM2*rs3730485) to explore the association between polymorphisms in cell-cycle genes and mtDNA copy number in coke oven workers. In this study, we demonstrated that rs1059234 and rs1801270 in *p21* have significant effect on the mtDNA copy number. The rs1059234 is a C-to-T transition that occurs 20 nucleotides downstream of the stop codon in 3′-untranslated region (UTR) of *p21* gene (35). Studies exhibited that the polymorphisms within 3′-UTR were associated with disease susceptibility (36). Accordingly, we found that the mtDNA copy number in the TT genotype in *p21* rs1059234 was statistically higher than that in the mutation homozygous CT+CC genotype in the exposed group. Previous research has shown that the 3′-UTR can affect mRNA degradation by regulating its nucleotide responsive region to interact with mRNA-binding protein, which eventually results in an alteration in protein expression levels (37, 38). It is likely that C-to-T transition in rs1059234 could increase *p21* mRNA expression by preventing degradation of mRNA, and thus possess a greater ability to control ROS levels in response to oxidative stress, ultimately reducing the loss of the mtDNA copy number caused by exposure to COEs. Moreover, the rs1801270 is a C-to-A transversion occurring in codon 31 of *p21* gene that causes an amino acid substitution from serine to arginine, which is expected to affect the DNA binding zinc finger domain of protein, eventually affecting expression and activity of *p21* (39). Our study showed that the rs1801270 was in strong linkage disequilibrium with the rs1059234, which suggests genotypes in the two loci are strongly correlated (35). Thus, the rs1801270 and the rs1059234 may have the same effect on the mtDNA copy number. Similar to the rs1059234, we found that the mutation homozygous AA genotype in rs1801270 had statistically higher mtDNA copy number than the CA+CC genotypes in coke oven workers.

Furthermore, we explored the interactions of COEs-exposure and gene on mtDNA copy number. It has shown that interactions of gene-environment play a determined role in many complex diseases (23). In this study, we found the significant combined effect of COEs-exposure and the CT+CC genotypes in *p21* rs1059234 on the mtDNA copy number, though the interaction was not statistically significant. Long-term exposure to PAHs could increase the level of ROS, and CT+CC genotype in *p21* rs1059234 also shows a lower ability to control ROS levels, both of which together lead to the gradual accumulation of ROS, which in turn cause mtDNA instability (40). In addition, the associations between polymorphisms in *p53* and *MDM2* genes and mtDNA copy number were not significant. This could be due to the environment-gene interaction, which may also cause alterations of mtDNA copy number.

In this study, we explored the association between mtDNA copy number and polymorphisms in cell-cycle genes compared to previous researches that mainly focused on the effect of nuclear genes on mtDNA copy number. Moreover, we further explored the combined effect of COEs-exposure and gene polymorphisms on mtDNA copy number in coke oven workers. However, several limitations need to be considered. First, we assessed the external COEs-exposure based on air concentration of COEs and working time, which could not represent the accurate individual exposure level. Second, the research only discovered the effects of the gene and COEs-exposure on mtDNA copy number, further experimental studies may be applied to elucidate the underlying mechanisms.

In conclusions, our study shows that COEs-exposure and the CT+CC genotypes in *p*21 rs1059234 are the major influencing factors of the mtDNA copy number. The findings can provide strong evidence for researching the mechanism of mtDNA copy number alterations caused by genetic and occupational factors and for screening effective predisposing biomarkers and early prevention of high-risk population.

## Data Availability Statement

The raw data supporting the conclusions of this article will be made available by the authors, without undue reservation.

## Ethics Statement

The studies involving human participants were reviewed and approved by Life Sciences of Institutional Review Board of Zhengzhou University Ethics, China. The patients/participants provided their written informed consent to participate in this study.

## Author Contributions

YY and XS: conceptualization. JT: formal analysis. WW: funding acquisition. XD: investigation. YW: writing—original draft. BL and LS: writing—review and editing. All authors have read and agreed to the published version of the manuscript.

## Funding

The work was supported by the National Science Foundation of China (NSFC81872597 and 81001239) and the HERCULES Exposome Research Centre (P30ES019776).

## Conflict of Interest

The authors declare that the research was conducted in the absence of any commercial or financial relationships that could be construed as a potential conflict of interest.

## Publisher's Note

All claims expressed in this article are solely those of the authors and do not necessarily represent those of their affiliated organizations, or those of the publisher, the editors and the reviewers. Any product that may be evaluated in this article, or claim that may be made by its manufacturer, is not guaranteed or endorsed by the publisher.
